# Integrated analysis of miRNAs and DNA methylation identifies miR‐132‐3p as a tumor suppressor in lung adenocarcinoma

**DOI:** 10.1111/1759-7714.13497

**Published:** 2020-06-04

**Authors:** Yun Su, Amol Shetty, Feng Jiang

**Affiliations:** ^1^ Department of Surgery Nanjing University of Chinese Medicine Nanjing China; ^2^ Institute for Genome Sciences University of Maryland School of Medicine Baltimore Maryland USA; ^3^ Department of Pathology University of Maryland School of Medicine Baltimore Maryland USA

**Keywords:** DNA methylation, epigenetics, lung cancer, microRNA

## Abstract

**Background:**

Aberrant miRNA expression and DNA methylation are two major epigenetic events in lung adenocarcinoma (LUAD). We conducted a combined analysis of the molecular changes in LUAD.

**Methods:**

We analyzed differentially expressed miRNAs and methylated CpG loci in 489 LUAD tissues versus 49 normal lung tissues of the Cancer Genome Atlas (TCGA). The results were validated in cell lines and xenograft mouse models and additional pairs of 36 LUAD and 36 normal lung tissues.

**Results:**

A total of 125 differentially expressed miRNAs and 145 differentially methylated CpG loci were identified in the LUAD versus normal lung tissues of TCGA data. Expression of the 22 miRNAs was inversely correlated with the 47 differentially methylated sites located in the miRNAs. Molecular and cellular function analysis showed that the abnormally methylated miRNAs were mainly involved in cell‐to‐cell signaling and interaction in airway cells. The DNA methylation status and altered expressions of miRNAs and their target genes were confirmed in 36 pairs of lung tumor and noncancerous lung tissues. Furthermore, aberrant miRNA expressions or DNA methylations alone could be involved in tumorigenesis of LUAD via different pathways. In addition, elevated miR‐132‐3p expression, reduced expression of its targeted gene (ZEB2), and decreased cell proliferation was observed in lung cancer cells treated with DNA methyltransferase inhibitor. Moreover, in vitro and in vivo analyses showed that miR‐132‐3p‐3p downregulation via DNA methylation promoted tumorigenicity of lung cancer by directly regulating ZEB2.

**Conclusions:**

The interaction between two epigenetic aberrations could have important functions in LUAD. miR‐132‐3p might act as a tumor suppressor in the tumorigenicity of LUAD.

**Key points:**

**Significant findings of the study:**

- Systemically investigating relationship between aberrant miRNA expression and DNA methylation in lung cancer could improve understanding of lung tumorigenesis and develop diagnostic and therapeutic targets.

**What this study adds:**

- Three forms of relationships between the two epigenetic changes are defined.
- miR‐132‐3p is further identified as a tumor suppressor in lung cancer.

## Introduction

Non‐small‐cell lung cancer (NSCLC) is the number one cancer killer in men and women. NSCLC mainly consists of three histological subtypes: lung adenocarcinoma (LUAD), squamous cell carcinoma (SCC), and large cell carcinoma (LCC). LUAD is the most common type of lung cancer, accounting for 40% of all NSCLCs. Molecular changes, particularly altered epigenetic processes, can lead to malignant cellular transformation.^1^ Through transcriptional silencing of critical cancer‐associated genes, DNA methylation plays a vital role in cancer pathogenesis.^1^ As another major component of epigenome, microRNA (miRNA) is a key regulator at the post‐transcriptional levels that modulate transcriptome changes.^2^ Furthermore, miRNAs regulate many cancer‐related genes associated with different biological processes, including proliferation, apoptosis, development, and tumorigenesis.^2^ Previous studies^3^, ^4^ have shown that altered methylation of miRNAs are associated with dysregulation of the genes and contribute to the development and progression of lung cancer.

The Cancer Genome Atlas (TCGA) contains multiomics data at different molecular levels and provides more than 10 000 patients with nearly 33 types of cancer.^5^ Integrated analysis of the multiomics data would deepen our understanding of the complex mechanisms underlying tumorigenesis.^5^ In this study, we investigated the epigenetic relationship between miRNA and DNA methylation in the pathogenesis of LUAD by analyzing TCGA multigenomic data. The results were further validated in cell lines and xenograft mouse models and additional LUAD tumor and the matched noncancerous lung tissues.

## Methods

### Data collection and data preprocessing

Genome‐wide DNA methylation, miRNA expression, and clinical information of the patients were retrieved from TCGA data portal (https: //tcga‐data.nci. nih.gov/tcga/, accessed March 2016). The inclusion criterion was the specimens with DNA methylation, miRNA expression, and clinical information. The platforms for DNA methylation were Human methylation 27 with 27 578 probes and Human Methylation 450 with 485 577 probes. The platforms for miRNA were Agilent two‐channel array with 722 human mature miRNA expressions and Illumina HiSeq_miRNASeq with 1036 human mature miRNA expressions. Finally, 489 LUAD tissues and 49 matched normal tissues were selected from 489 lung cancer patients. The detailed clinical characteristics of the patients are listed in Table 1. To analyze miRNA and DNA methylation data, we removed outlier samples with TCGAanalyze_Preprocessing function of TCGAbiolinks using a Spearman correlation cutoff of 0.6. The datasets for GC‐content [47] and library size were normalized by using the TCGAanalyze_Normalization. The miRNA data between LUAD and normal tissues were analyzed using limma package in R. The fold changes (FCs) in the expression of individual miRNAs were calculated and miRNAs with log2 (FC) >1.0 and *P* < 0.05 were significant. We used the MEDIPS package (version 1.24.0) for the analysis and comparison of DNA methylation datasets of LUAD and normal lung tissues.^6^ *P*‐values <0.05 and log2 (FC) ≥1 were considered to show differentially methylated CpG island loci.

**Table 1:** Clinical characteristics of 489 patients diagnosed with LUAD in TCGA

	Number of cases (%)
Age at diagnosis	
<60	133 (27.2%)
>60	356 (72.8%)
Sex	
Female	196 (40.1%)
Male	293 (59.9%)
Smoking status	
10 < Pack‐years	222 (45.4%)
10 ≥ Pack‐years	221 (45.2%)
NA	46 (9.4%)
Stage	
Stage I	340 (73.0%)
Stage II	73 (14.9%)
Stage III–IV	58 (12.1%)

LUAD, lung adenocarcinoma; TCGA, the Cancer Genome Atlas.

### Lung tissue specimens

With a study protocol (Protocol number: HP00040666) approved by the Institutional Review Board of the University of Maryland Baltimore, we obtained 36 frozen LUAD tumor tissues and the matched noncancerous lung tissues from a tissue bank of the University Medical Center. We reviewed complete medical records of the patents. Clinical diagnosis of lung cancer was made using histopathologic examinations of specimens obtained by CT‐guided transthoracic needle biopsy, transbronchial biopsy, video‐assisted thoracoscopic surgery (VATS), or surgical resection. The surgical pathologic staging was determined according to the TNM classification of the International Union Against Cancer with the eighth American Joint Committee on Cancer and the International Staging System for Lung Cancer. Histopathological classification was determined according to the World Health Organization classification. Clinical characteristics of the surgically‐resected tissues are shown in Table 2.

**Table 2:** Clinical characteristics of surgically‐resected lung tumor diagnosed with LUAD and matched noncancerous normal lung tissues of 36 patients

Age	66 (SD 9.4)
Sex	
Female	10
Male	26
Smoking status	
Pack‐years	30.3 (SD 9.2)
Stage	
Stage I	24
Stage II	6
Stage III‐IV	6

LUAD, lung adenocarcinoma; SD, standard deviation.

### Analysis of DNA methylation in miRNAs

We extracted the transcription start site (TSS) data of miRNAs from the Functional Annotation of the Mammalian Genome5 database and tMethyl‐CpG binding domain‐sequencing data. We considered 1000 bp upstream and downstream as coding sequence regions of miRNAs.^7^

### Analysis of DNA methylation by quantitative methylation specific PCR (qMSP)

We performed DNA isolation and qMSP as described in our previous publications.^8^, ^9^ Briefly, we extracted DNA from the specimens using DNeasy kit (Qiagen, Valencia, CA). We eluted DNA with 50 μL of elution buffer (10 mmol/L Tris‐Cl, pH 8.5) (Sigma‐Aldrich Corporation). DNA was quantified using the Quantifiler Human DNA Quantification kit (Applied Biosystems, Foster City, CA). Bisulfite conversion was carried out on DNA using the Zymo EZ DNA Methylation Kit (Zymo Research, Irvine, CA) according to the manufacturer's protocol. qMSP was done by using a Lightcycler system (Roche Applied Science, Mannheim, Germany)^10^. Hypermethylation and hypomethylation markers for the genes were designed. Cycle threshold (Ct) values for each gene were determined. We normalized Ct values of the target genes in relation to that of myogenic differentiation antigen 1 (MYOD1), which was used as control for the qMSP analysis.^8^, ^9^ By using the formula: 2∧(Ct (MYOD1) − Ct (target gene) × 100, we computed ratio value to decide the relative level of methylation of the genes in each sample as previously described.^10^

### Analysis of expressions of the miRNAs and the targeted genes by using quantitative reverse transcription PCR (RT‐qPCR)

We extracted RNA by using a protocol established in our previous reports.^2^ The purity and concentration of RNA were determined from OD260/280 readings by using a dual‐beam Ultraviolet (UV) spectrophotometer (Eppendorf AG, Hamburg, Germany). RNA integrity was determined by capillary electrophoresis by using the RNA 6000 Nano Lab‐on‐a‐Chip kit and the Bioanalyzer 2100 (Agilent Technologies, Santa Clara, CA). We evaluated the expressions of miRNAs and the targeted genes by qRT‐PCR with Taqman miRNA assays (Applied Biosystems, Foster City, CA).^2^ Briefly, RNA was applied for reverse transcription (RT) by using the Applied Biosystems 9700 Thermocycler (Applied Biosystems) with miRNA‐specific looped primer and TaqMan MicroRNA RT Kit (Applied Biosystems). The reaction included 50 nM stem‐loop RT primer, ×1 RT buffer, 0.25 mM each of deoxyrinonucleotides, and 3.33 U/μL MultiScribe reverse transcriptase in a total volume of 15 μL. The 20 μL PCR reaction included RT product, ×1 TaqMan Universal PCR Master Mix (Applied Biosystems), and the corresponding primers and Taqman probe for the target genes. The reactions were incubated in a 94 well plate at 95°C for 15 minutes, followed by 45 cycles of 95°C for 15 seconds and 60°C for one minute. We normalized Ct values of the target miRNAs or the targeted genes in relation to that of U6 and determined relative expression of a gene in a given sample using the eq. 2 − ΔCt, where ΔCt = Ct (targeted genes) − Ct (U6). U6 has been considered one of the suitable endogenous controls for the quantification of miRNA and protein‐coding genes in tissue specimens.^11^ Two interplate controls and one no‐template control were carried along in each experiment. All experiments were performed at least three times.

### Lentivirus infection

Recombined LV‐GV369‐miR‐132‐3p‐EGFP and the respective negative control vector were commercially manufactured by GeneChem Co, Ltd. (Shanghai, China). We transduced the lentiviral vectors into A549 cells (ATCC, Manassas, VA) as described in our previous studies.^12^, ^13^, ^14^, ^15^, ^16^, ^17^, ^18^ Briefly, we seeded (2 × 10^5^ cells/mL) in 6 well plates and incubated the cells for 24 hours to reach 50% confluence, which were replaced with infection medium containing lentiviral vectors at a multiplicity of infection (MOI) of 20 plaque‐forming units/cell. Successfully infected cells were GFP positive under a fluorescent microscope after 48 hours. We evaluated efficiency of miR‐132‐3p overexpression by PCR.

### Western blot analysis

We performed western blotting as described in our previously studies^12^, ^14^, ^17^ with antibodies against ZEB2 or NOVA1 (Santa Cruz Biotechnology, Inc., Santa Cruz, CA) or β‐actin (Sigma‐Aldrich Co., St Louis, MO).

### Treatment of cancer cells with 5‐aza‐2′‐deoxycytidine and miRNA inhibitor

Two human LUAD cell lines (A549 and H1975) were obtained from the ATCC. We seeded the cell lines at a density of 5 × 10^6^/well in six‐well plates. The cells were cultured for 24 hours and treated with 10 μM of the DNA demethylation agent 5‐aza‐2′‐deoxycytidine (5‐Aza‐CdR; Sigma‐Aldrich Co) and phosphate buffered saline (PBS) (Sigma‐Aldrich Co., St Louis, MO), respectively. The cells were also treated with either 200 nmol/L of miR‐132 inhibitor or inhibitor negative control (Thermo Fisher Scientific Inc., Halethorpe, MD), with Lipofectamine 2000 reagent, respectively (Thermo Fisher Scientific Inc.), according to the manufacturer's instructions. Transfection efficiencies were determined by qRT‐PCR.

### Cell proliferation assay

Cell proliferation was performed using the methylthiazol tetrazolium (MTT) assay as described in our previous studies.^14^, ^16^, ^18^ Briefly, the cells were counted and seeded in six‐well plates at a density of 1000 cells per well after transfection. Cells were allowed to grow for 24, 36, and 72 hours. The cells were then stained using crystal violet and each colony with a minimum of 50 cells and counted at each time point. All experiments were repeated three times, each sample in triplicate. Data represented the mean (± SD) of three independent experiments.

### Cell cycle analysis by flow cytometry

DNA content was analyzed by using flow cytometry as previously described.^14^, ^18^ Briefly, harvested cells were washed twice with 5mM EDTA/PBS and fixed with 95–100% cold ethanol and kept at 4°C overnight. Cells were incubated with RNase A (50 μg/mL), and then stained with propidium iodide (PI, 10 mg/mL) (Sigma) for DNA content analysis on a FACScan flow cytometer (Becton Dickinson). Statistical analysis of variance (ANOVA) and least significant difference *t*‐tests were used to compare cell cycle data of cells with and without specific siRNA treatments. All experiments were repeated three times, each sample in triplicate. Data represented the mean (± SD) of three independent experiments.

### Transmigration and wound healing assays

Cells were plated in medium without serum in the top chamber of a transwell (Corning, NY) in order to determine migration and invasion. The bottom chamber contained standard medium with 10% FBS. After incubation for 24 hours, the cells that had migrated to the lower surface of the membrane were fixed with formalin and stained with crystal violet. The migrating cells were examined microscopically and determined by counting the migrating/invasive cells in five randomly selected fields using an Olympus BX41 microscope. Photomicrographs were taken using a Qcolor5 digital camera system fitted to this microscope. To determine whether the inhibition of cell migration by forced expression of miR‐132‐3p was due to the inhibition of cell proliferation, the cells with forced expression of miR‐132‐3p were treated with aphidicolin (1 mg/mL) (Sigma‐Aldrich), a proliferation inhibitor. A wound healing assay was performed in 12 well plates (1 × 10^5^ per well). When the cells were grown to 90%–95% confluences, transfection of cancer cells were performed. Wound lines were created manually by scratching the monolayer with a sterile 200 ml pipette tip and migration of the cells was assessed after 24, 48, and 72 hours. Pictures were taken using a Nikon inverted phase‐contrast microscope (Nikon, Melville, NY). The distance between the parallel lines was measured using ImageJ software. All experiments were carried out at least three times. All experiments were repeated three times, each sample in triplicate. Data represented the mean (± SD) of three independent experiments.

### Tumorigenicity assays in nude mice

The animal study was performed with the approval of the University of Maryland Baltimore under code IACUC# 0516007. Briefly, five athymic Balb/c, Nu/Nu mice per group were inoculated with 1 x 10^6^ A549 cells infected with LV‐GV369‐miR‐132‐3p‐vector and 1 × 10^6^ A549 cells with control vector, respectively. All the animals were monitored regularly, and tumor growth was measured at regular intervals. The mice were observed for four weeks and then euthanized under deep anesthesia with pentobarbital (Sigma). We calculated volume of the tumors by using formula (length [mm]) × (width [mm]) 2 × 0.52. The tumor size was represented by mean ± SD mm^3^.

### Pathway analyses

We use Ingenuity Pathway Analysis (IPA) (Ingenuity Systems, Mountain view, CA) to identify the most significant pathways of the genes, as previously described.^19^ The gene list files containing gene symbols, FC, *P*‐values were uploaded to IPA for the analysis.

### Statistical analysis

All data were expressed as mean ± standard deviation (SD). We used unpaired *t*‐test to analyze expression levels of miRNAs and methylation status in LUAD and corresponding normal tissues. We used chi‐square analysis and Pearson's correlation test to evaluate correlation between methylation status and expression levels of miRNAs. We performed the chi‐square and *t*‐tests to evaluate the relationship between the molecular changes and clinical features. A *P*‐value of less than 0.05 was considered statistically significant.

## Results

### Differentially expressed miRNAs in LUAD tissues versus normal lung tissues

Multiomics data of 489 LUAD and 49 matched noncancerous lung tissues of lung cancer patients in the TCGA were analyzed. The detailed clinical characteristics of the patients are listed in Table 1. From the cohort of the TCGA data, we identified a total of 125 miRNAs that were differentially expressed between the LUAD tissues and matched normal tissues (*P* < 0.05 and FC > 1.0) (Table S1). A total of 89 miRNAs were significantly upregulated and 36 were downregulated in the LUAD tissues versus normal lung tissues. Furthermore, we obtained 36 frozen LUAD tumor tissues and the matched noncancerous lung tissues from a tissue bank of the University Medical Center (Table 2). We selected eight miRNAs (five upregulated and three downregulated miRNAs) and used qRT‐PCR to validate the expression levels in the 36 LUAD tissues and the 36 matched normal lung tissues. The eight miRNAs exhibited a significantly different level in the LUAD tissues compared with normal lung tissues (all *P* < 0.05) (Table 3). Moreover, the eight miRNAs had changes in the tissue specimens by qRT‐PCR in the same direction as by deep sequencing analysis in TCGA. Therefore, the results generated from the independent set of lung tumor and noncancerous lung tissues confirmed the findings from TCGA data.

**Table 3:** Expression of eight miRNAs in stage I adenocarcinoma and normal lung tissues measured by RT‐PCRMiRNAs

	Mean ± SD in normal tissues	Mean ± SD in adenocarcinoma tissues	*P*‐value
miR‐577	0.234 51 ± 0.0242345	0.785 15 ± 0.021453	0.006564
miR‐196a‐5p	2.435 54 ± 0.746 75	11.5645 ± 2.574	0.0006546
miR‐182‐3p	0.04546 ± 0.01356	0.123 454 ± 0.02446	0.0067434
miR‐147b	0.223 576 ± 0.1232	0.542 343 ± 0.03454	0.00054534
miR‐183‐3p	3.476 ± 0.2574	6.2554 ± 1.4545	0.00956545
miR‐184	4.3451 ± 0.564 34	0.234 354 3 ± 0.042352	0.00012436
miR‐486‐5p	212.29 ± 32.46	17.23 ± 8.2874	2.69E‐03
miR‐30a‐3p	327.48 ± 36.2538	22.16 ± 5.028	4.57E‐07

RT‐PCR, reverse transcription‐polymerase chain reaction; SD, standard deviation.

### Differentially methylated loci in LUAD tissues versus normal lung tissues

**Figure 1:**
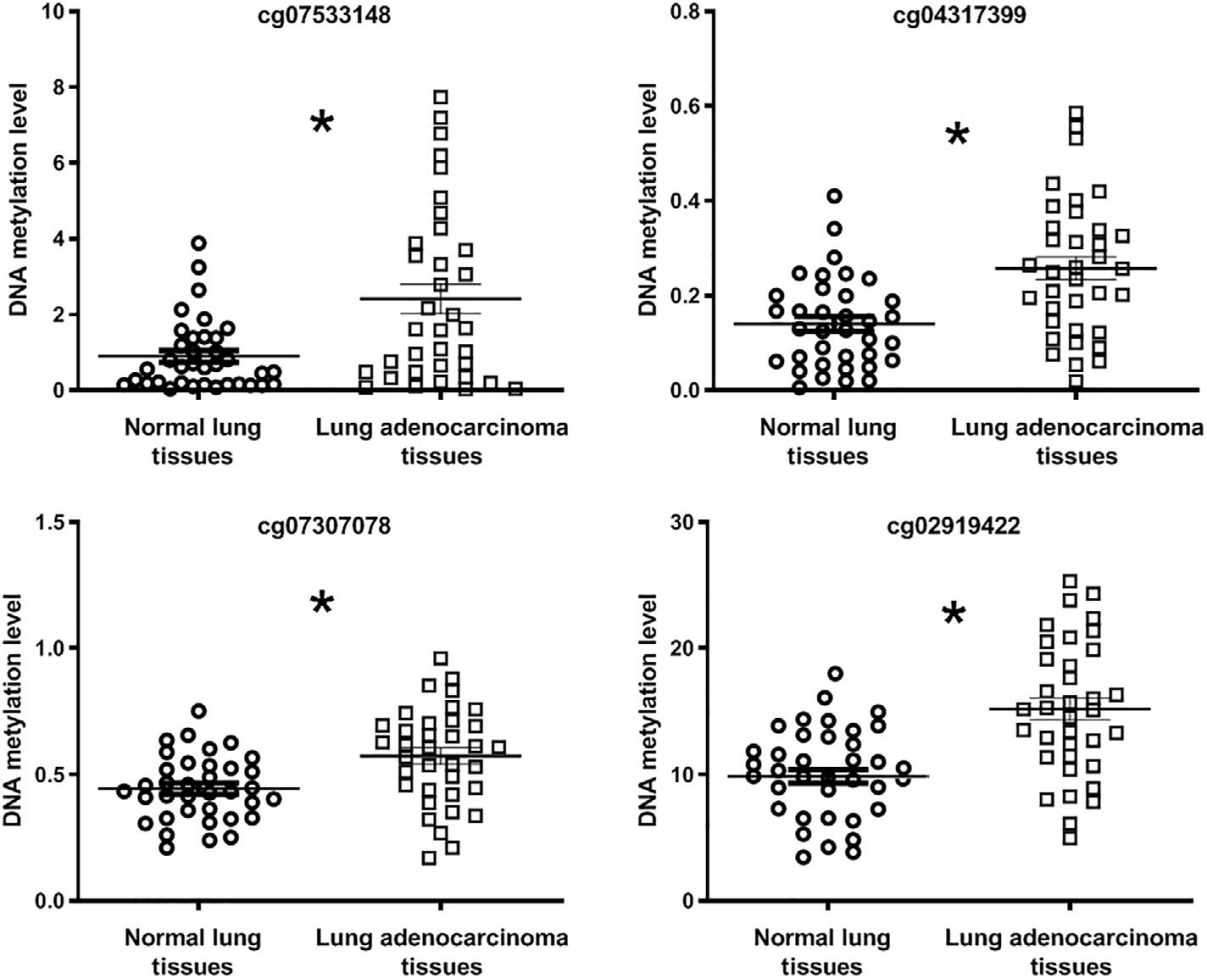
Quantitative methylation specific PCR analysis of four differentially methylated loci in 36 lung adenocarcinoma tissues and 36 normal lung tissues confirmed data from the TCGA database. All four methylation loci (cg07533148, cg04317399, cg07307078, and cg02919422) were hypermethylated in lung adenocarcinoma tissues compared with the matched normal lung tissues (*all *P* < 0.05).

A total of 145 CpG‐islands were differentially methylated in the 489 LUAD tissues versus 49 noncancerous lung tissues (Table S2). Of the methylation loci, 110 and 35 were hypermethylated and hypomethylated in tumor samples, respectively, compared with normal lung tissues (all *P* < 0.05). We tested four loci (cg07533148, cg04317399, cg07307078, and cg02919422) for the methylation status in additional 36 LUAD tissues and the matched normal lung tissues. The four methylated loci had the same changes in the tissue specimens as in TCGA data (Fig *1*). Therefore, the findings produced from the different tissue specimens validated the discoveries from TCGA data.

### Relationship between miRNA and DNA methylation in LUAD

To identify miRNA‐DNA methylation site pairs where DNA methylation sites were located within 1000 bps from the miRNA upstream and downstream regions, we extracted the TSS data of miRNAs from the FANTOM5 database. We then used Spearman correlation and MA to analyze the correlation between DNA methylation and miRNA expression. Of the 125 differentially expressed miRNAs, 22 (17.6%) were inversely correlated with differentially methylated loci within 1000 bps from the miRNA upstream and downstream regions (Table 4) (all *P* < 0.05). Of the 145 differentially methylated loci, 47 (32.4%) had opposite association with the 22 miRNAs (Table 4) (all *P* < 0.05).

**Table 4:** Epigenetic interactions between miRNAs and in lung adenocarcinoma

miRNAs	Expression level in tumors	Site ID	Methylation	*P*‐value	Distance	Location in miRNA
mir‐1236	Down	cg21754201	Hyper	0.0108	−222	Downstream
		cg25355006	Hyper	0.0040	−94	Downstream
mir‐1276	Down	cg04091325	Hyper	0.0060	−702	Downstream
mir‐132	Down	cg08457620	Hyper	<0.0001	−179	Downstream
mir‐143	Down	cg17736336	Hyper	0.0120	273	Upstream
mir‐147b	Over	cg23803468	Hypo	0.0010	−964	Downstream
mir‐200a	Over	cg07213830	Hypo	<0.0001	−834	Downstream
		cg14288281	Hypo	0.0010	−351	Downstream
mir‐200b	Over	cg20256117	Hypo	0.0120	−988	Downstream
mir‐219‐1	Down	cg02538046	Hyper	0.0190	−757	Downstream
		cg03000593	Hyper	0.0010	428	Upstream
		cg04678230	Hyper	<0.0001	401	Upstream
		cg07245868	Hyper	0.0010	535	Upstream
		cg08771019	Hyper	0.0030	445	Ustream
		cg10134527	Hyper	<0.0001	575	Upstream
		cg13027595	Hyper	<0.0001	406	Upstream
		cg14096569	Hyper	<0.0001	526	Upstream
		cg14258935	Hyper	0.0080	304	Upstream
		cg14288848	Hyper	0.0230	717	Upstream
		cg18144560	Hyper	<0.0001	520	Upstream
		cg20185718	Hyper	0.0220	273	Upstream
		cg21330831	Hyper	<0.0001	498	Upstream
		cg25954512	Hyper	<0.0001	569	Upstream
		cg26646118	Hyper	<0.0001	590	Upstream
		cg27168291	Hyper	<0.0001	593	Upstream
		cg27368379	Hyper	0.0020	262	Upstream
mir‐339	Down	cg12195211	Hyper	0.005	23	Upstream
mir‐375	Over	cg01717376	Hypo	<0.0001	−234	Downstream
mir‐429	Over	cg07718444	Hypo	<0.0001	168	Upstream
mir‐516a‐1	Over	cg11618529	Hyper	0.0300	−301	Downstream
mir‐548d‐1	Over	cg12646585	Hypo	0.0080	38	Upstream
		cg14018100	Hypo	0.0030	545	Upstream
mir‐590	Over	cg22123387	Hypo	0.0190	−272	Downstream
mir‐625	Over	cg13210403	Hypo	0.0080	885	Upstream
		cg19619721	Hypo	0.0170	512	Upstream
mir‐675	Over	cg02928928	Hypo	<0.0001	−739	Downstream
		cg07435282	Hypo	0.0030	−671	Downstream
		cg14836313	Hypo	0.0250	−995	Downstream
		cg19273253	Hypo	0.0070	433	Upstream
		cg19837124	Hypo	<0.0001	398	Upstream
mir‐744	Down	cg25237720	Hyper	0.0010	900	Upstream
mir‐877	Down	cg16979445	Hyper	0.0040	445	Upstream
mir‐9‐2	Over	cg17224769	Hypo	0.0020	526	Upstream
mir‐92b	Over	cg02892624	Hypo	0.0030	−536	Downstream
		cg17421062	Hypo	0.0040	−462	Downstream
mir‐937	Over	cg10817223	Hypo	0.0020	169	Upstream
mir‐940	Down	cg07032866	Hyper	0.0050	979	Upstream

There were more than one differentially methylated sites located in either upstream or downstream regions of miRNAs (Table 4). The 22 miRNAs might be epigenetically regulated by the DNA methylation in the genes. Of the 22 miRNAs, nine miRNAs were downregulated by hypermethylation, and 13 miRNAs were upregulated by the hypomethylation. However, of the 125 differentially expressed miRNAs, 103 (82.4%) were not associated with altered DNA methylation (Table S3). Furthermore, of 187 differentially methylated loci in LUAD tissues, 98 (67.6%) were not associated with expression of the miRNAs (Table S4).

To investigate if the direct interaction between miRNA and DNA methylation could affect expression of target genes of the miRNAs, we used miRNA enrichment to predict potential target genes of miRs‐132‐3p and ‐339. ZEB2 and NOVA1 were the top predicted genes of the two miRNAs, respectively.

**Figure 2:**
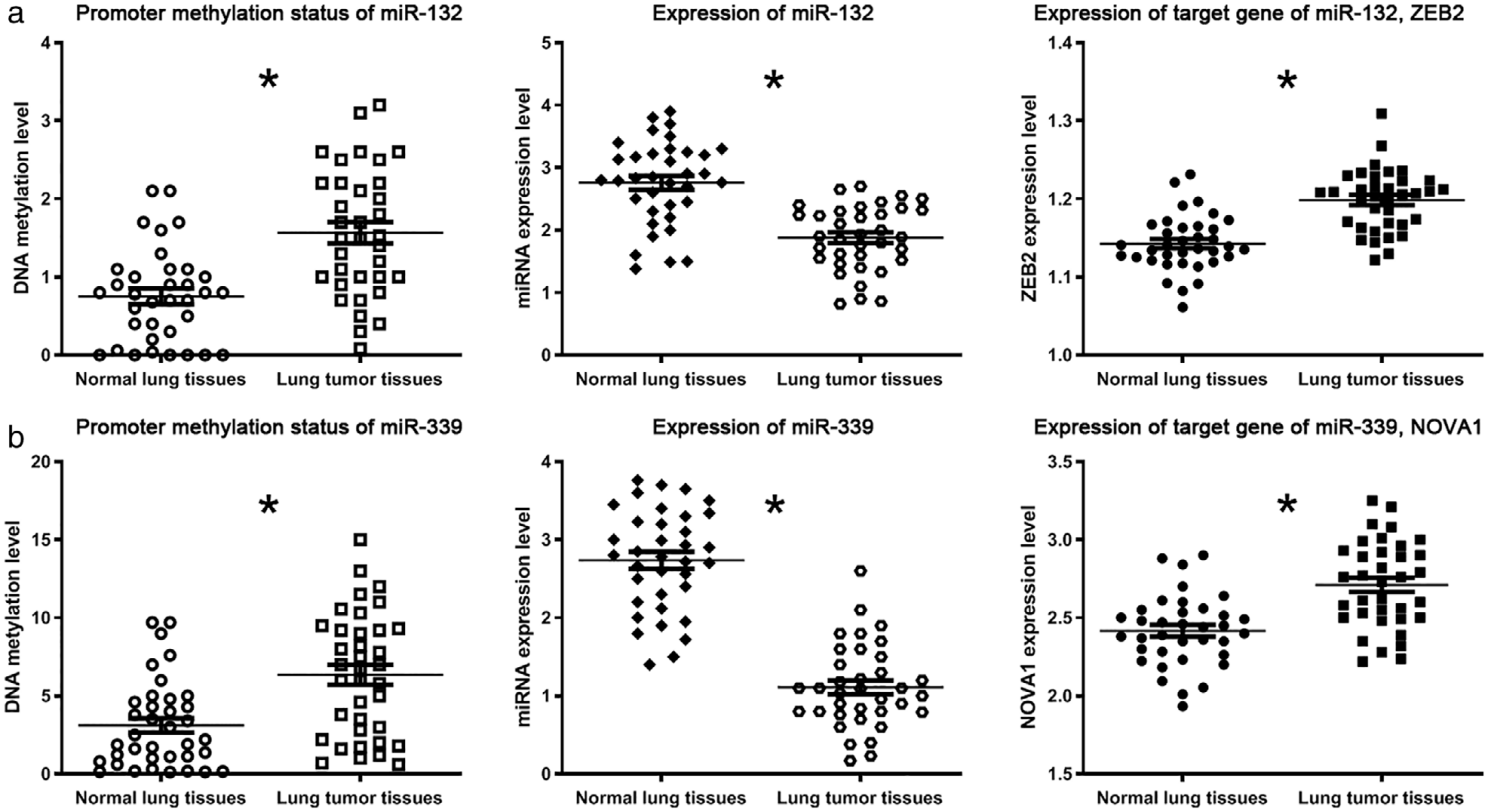
Quantitative methylation specific PCR analysis of methylated loci of miRs‐132 and 339 and RT‐qPCR analysis of the miRNAs in the 36 lung adenocarcinoma tissues and 36 normal lung tissues. (**a**) miR‐132‐3p was significantly hypermethylated and downregulated in lung adenocarcinoma tissues compared with the matched normal lung tissues. However, the target gene of miR‐132‐3p, ZEB2, was overexpressed in the paired lung adenocarcinoma tissues versus normal lung tissues. (**b**) miR‐339 was considerably hypermethylated and downregulated in lung adenocarcinoma tissues versus the matched normal lung tissues. However, the target gene of miR‐339, NOVA1, was overexpressed in the paired lung adenocarcinoma tissues versus normal lung tissues (*all *P* < 0.05).

We then validated the bioinformatic findings in the 36 LUAD tissues and 36 normal lung tissues. As shown in Fig 2, hypermethylation and reduced expression of miRs‐132 and ‐339 and elevated expression of the target genes were validated in the LUAD tissues compared with normal lung tissues. Therefore, the interaction between the two epigenetic events and their impact on their target genes were experimentally validated.

### Inhibiting DNA methylation in LUAD cells could elevate miR‐132‐3p expression and reduce expression of the target gene

To confirm whether expression of the miR‐132‐3p was affected by the methylation status, we treated two LUAD cell lines (A549 and H1975) with 5‐Aza‐dC. In silico prediction of CpG islands of miR‐132‐3p and the DNA methylation levels in LUAD cells are shown in Fig . The A549 cancer cells treated with 5‐Aza‐dC had a signifcantly lower methylation level compared with the cells without the treatment (*P* = 0.00016). Furthermore, 5‐Aza‐dC treatment could elevate the expression of miR‐132‐3p in the two cell lines (Fig *4*a). In addition, the 5‐Aaz‐dC treatment suppressed the expression level of ZEB2 (Fig *4*b), a direct target gene of miR‐132‐3p. Moreover, cell proliferation was reduced in the cancer cells treated with 5‐Aza‐dC compared to cancer cells treated with PBS (Fig *4*c). However, miR‐132‐3p inhibitor increased cell proliferation of the cancer cells (Fig *4*c). Furthermore, the effect of 5‐Aza‐dC on reduction of cell proliferation was significantly diminished by miR‐132‐3p inhibitor. Therefore, the hypermethylation of the miRNAs could regulate expression of the miRNAs, which in turn, upregulated the corresponding targeted genes, and cell proliferation.

**Figure 3:**
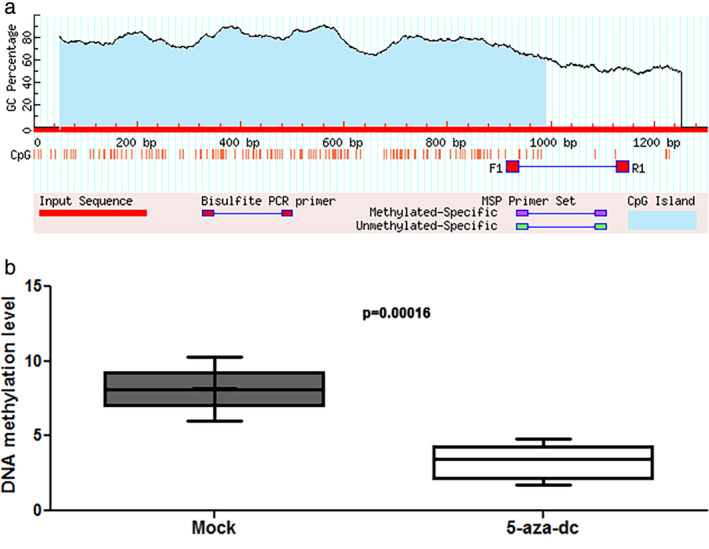
In silico prediction of CpG islands of miR‐132‐3p and the DNA methylation levels in LUAD cells treated with or without 5‐Aza‐dC. (**a**) CpG islands in miR‐132‐5p and location of the primers for qMSP. (**b**) the A549 cancer cells treated with 5‐Aza‐dC had a low methylation level compared with the cells without the treatment (*P = 0.00016).

**Figure 4:**
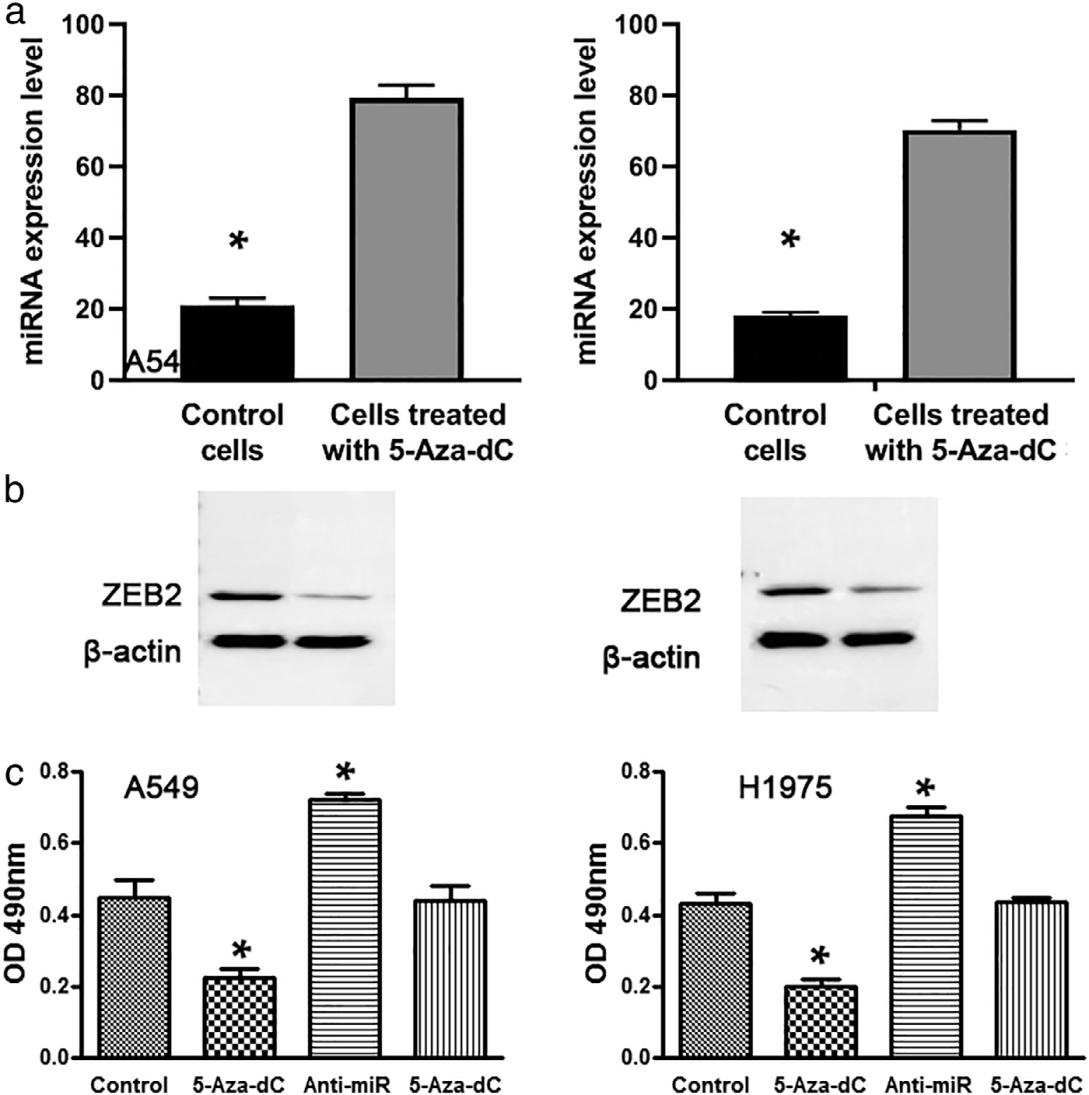
The treatment with 5‐Aza‐dC increased the expression level of miR‐132‐3p and decreased the level of ZEB2 protein and cell proliferation in two lung cancer cell lines, A549 and H1975. The cells were treated with 5 μM 5‐Aza‐dC. Each sample was subjected to (**a**) qRT‐PCR for detection of miR‐132‐3p. (**b**) Western blot for protein expression of ZEB2. (**c**) Cell proliferation was performed on the cells treated with 5‐Aza‐dC or miR‐132‐3p inhibitor or their combination for 48 hours, respectively. Cell proliferation was reduced or increased in the cancer cells treated with 5‐Aza‐dC or miR‐132‐3p, respectively, compared with cancer cells treated with PBS (*, all *P* ≤ 0.05). However, the effect of 5‐Aza‐dC on cell proliferation was counteracted by miR‐132‐3p inhibitor.

### miR‐132‐3p could reduce in vitro tumorigenicity of LUAD cells

**Figure 5:**
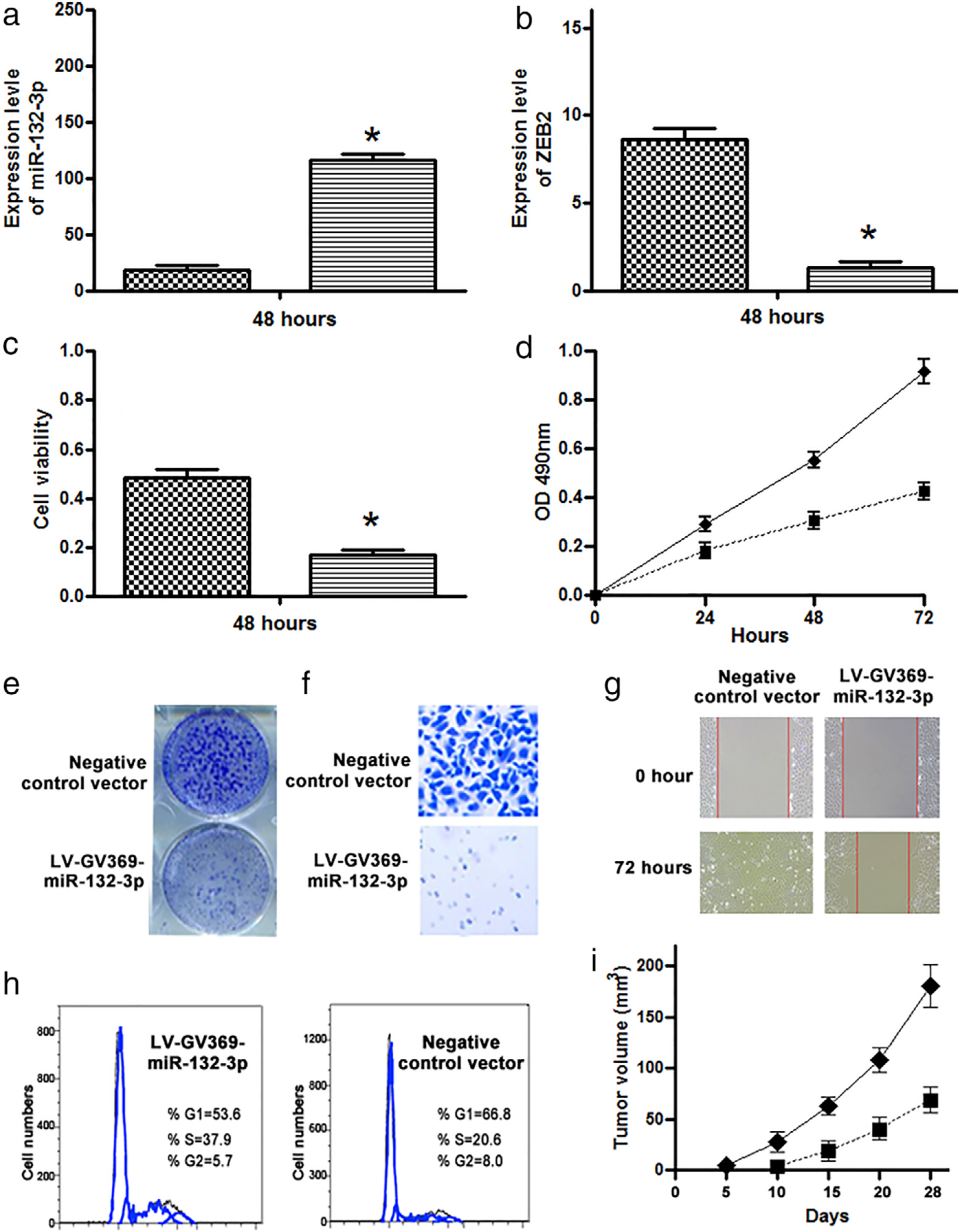
Forced expression of miR‐132‐3p could inhibit the tumorigenicity of A549 lung cancer cells. (**a**) Expression level of miR‐132‐3p was significantly higher in cells with ectopically expressed miR‐132‐3p versus negative controls at 48 hours. (**b**) Ectopic expression of miR‐132‐3p reduced the expression level of ZEB2, a direct target gene of miR‐132‐3p. (**c**) Forced expression of miR‐132 can significantly reduce cell viability. (**d**) Cells with ectopic expression of miR‐132‐3p had a lower cell proliferation comprised with cells with negative control vector. (**e**) Forced expression of miR‐132‐3p decreased cell colony formation. (**f**) Transwell migration assay shows that forced expression of miR‐132‐3p could constrain migration and invasion of A549 cancer cells. (**g**) In the wound‐healing assays, cancer cells with forced expression of miR‐132‐3p showed a slower gap closure compared with cells transfected with control vector. The figure only shows the results of A549 cells from the time points 0 and 72 hours, respectively. (**h**) Forced expression of miR‐132‐3p in lung cancer cells elevated the percentage of the cancer cells in the G1/G0 phase and reduced the percentage of S phase (*all *P* < 0.05). (**i**) Measurement of the tumor size over the time in vivo experiment shows that tumor growth in the mice injected with cancer cells with forced expression of miR‐132‐3p was significantly lower than in the mice injected with the cells transfected with control vector. Tumor sizes (mean ± SD^3^) were measured at the indicated intervals and plotted. (**a**) (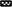) Negative controls vector, (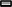) LV‐GV369‐miR‐132‐3p; (**b**) (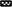) Negative controls vector, (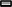) LV‐GV369‐miR‐132‐3p; (**c**) (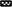) Negative controls vector, (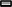) LV‐GV369‐miR‐132‐3p; (**d**) (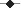) Negative controls vector, (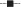) LV‐GV369‐miR‐132‐3p; (**i**) (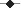) Negative controls vector, (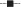) LV‐GV369‐miR‐132‐3p.

A549 cells with ectopically expressed miR‐132‐3p had more than 85% transfection efficiency at 48 hours. Expression level of miR‐132‐3p was significantly higher in cells with ectopically expressed miR‐132‐3p compared with cells with negative controls at 48 hours (Fig 5a). Furthermore, the expression level of ZEB2, a direct target gene of miR‐132‐3p, was considerably lower in the cells with ectopically expressed miR‐132‐3p compared with cells with control vector (Fig 5b). In addition, forced expression of miR‐132‐3p could reduce cell viability compared to cells with control vector (Fig 5c). The cells with ectopic expression of miR‐132‐3p had a lower cell proliferation comprised with cells with negative control vector (Fig 5d). Moreover, forced expression of miR‐132‐3p in lung cancer decreased their colony formation, migration, and invasion (Fig 5e–g). Finally, forced expression of miR‐132‐3p induced the percentage of cells in the G1/G0 phase by more than 10%, whereas it decreased the percentage of S phase at least 10% compared with cancer cells with a lower level of the miRNA (all *P* ≤ 0.05) (Fig 5h).

### miR‐132‐3p could reduce in vivo tumorigenicity of LUAD cells

Tumors were visible as early as six days in the mice that received negative control vector cancer cells. On 15 days post‐injection, tumors appeared in all the five mice injected with the A549 cells with negative control vector, whereas they were observed in only two of the five mice injected with ectopically expressed miR‐132‐3p. Furthermore, the tumors generated from the A549 cells with negative control vector were significantly larger compared to those produced from the cancer cells with ectopically expressed miR‐132‐3p at the end of observation (28 days) (180.33 ± 21.36 mm^3^ vs. 68.67 ± 13.01 mm^3^, *P* = 0.002) (Fig 5i). These in vivo findings in ectopic xenograft mouse models are consistent with the in vitro observations, and hence support that miR‐132‐3p could function as a tumor suppressor tumorigenicity of lung cancer.

### Bioinformatic molecular and cellular functional and pathway analyses

To better understand the biological function of the relationships between the two epigenetic evens in lung carcinogenesis, we used Ingenuity Pathway Analysis (IPA) to identify possible regulatory networks, by which the miRNAs with altered DNA methylation might be involved in LUAD. The 22 miRNAs with abnormal hypermethylation were mostly associated with molecular and cellular functions, including cell‐to‐cell signaling and interaction, cellular compromise, cellular development, cellular growth and proliferation, and cell cycle (Table 5). Furthermore, the highest enriched pathways of the miRNAs were IL‐17A signaling in airway cells, PEDF signaling, Wnt/β‐catenin signaling, and JAK family kinases in IL‐6‐type cytokine signaling, and role of macrophages, fibroblasts and endothelial cells in rheumatoid arthritis (Table 5).

**Table 5:** Molecular and cellular functional and pathway analyses

	Molecular and cellular functions	*P*‐value	Canonical pathways	*P*‐value
The 22 miRNAs with abnormal hypermethylation	1) Cell‐to‐cell signaling and interaction	0.00927	1) IL‐17A signaling in airway cells	0.00155
	2) Cellular compromise	0.0135	2) PEDF signaling	0.00315
	3) Cellular development	0.0018	3) Wnt/β‐catenin signaling	0.00342
	4) Cellular growth and proliferation	0.0036	4) Role of JAK family kinases in IL‐6‐type cytokine signaling	0.00357

	5) Cell cycle	0.0238	5) Role of macrophages, fibroblasts and endothelial	5.86E‐05
			cells in rheumatoid arthritis	
The 103 differentially expressed miRNAs, which are not associated with the differentially methylated loci	1) Cellular movement	2.33E‐06	1) Glucocorticoid receptor signaling	8.59E‐06
	2) Cellular assembly and organization	6.56E‐06	2) Glioblastoma multiforme signaling	1.38E‐05
	3) Cellular function and maintenance	3.23E‐05	3) Hepatic fibrosis/hepatic stellate cell activation	2.53E‐05
	4) Cellular development	7.66E‐05	4) Myc mediated apoptosis signaling	5.86E‐05
	5) Cellular growth and proliferation	0.000119	5) Estrogen‐mediated S‐phase entry	6.14E‐05
The 98 differentially methylated loci, differentially expressed miRNAs	1) Cellular development	0.000402	1) Glutamate receptor signaling	0.000227
	2) Cellular growth and proliferation	0.00228	2) CREB signaling in neurons	0.000783
	3) Cellular function and maintenance	0.00271	3) Amyotrophic lateral sclerosis signaling	0.0017
	4) Cell morphology	0.00269	4) Gap junction signaling	0.0039
			5) Gαi signaling	0.00425

The 98 differentially methylated loci, which were independent of the DNA methylation/miRNA/mRNA (protein) axis, were assigned to molecular and cellular functions, cellular development, cellular growth and proliferation, cellular function and maintenance, and cell morphology (Table 5). Furthermore, the differentially methylated loci were highly associated with canonical pathways of glutamate receptor signaling, CREB signaling in neurons, amyotrophic lateral sclerosis signaling, gap junction signaling, and Gαi signaling (Table 5).

The 103 differentially expressed miRNAs not epigenetically regulated by DNA methylation, mainly have molecular and cellular functions of cellular movement, cellular assembly and organization, cellular function and maintenance, cellular development, and cellular growth and proliferation (Table 5). The differentially expressed miRNAs might play important roles in LUAD through pathways of glucocorticoid receptor signaling, glioblastoma multiforme signaling, hepatic fibrosis/hepatic stellate cell activation, myc mediated apoptosis signaling, and estrogen‐mediated s‐phase entry (Table 5).

Therefore, there might be three forms of relationships between the two epigenetic events in tumorigenesis of LUAD: (i) synergetic interaction of microRNAs and DNA methylation contribute to the tumorigenesis by the DNA methylation/miRNA/mRNA (protein) axis. (ii) dysregulation of miRNAs; or (iii) abnormal DNA methylation alone might be involved in LUAD development and progression via different pathways, respectively.

## Discussion

Investigating the relationship of the DNA methylation and altered expressions of miRNAs could improve our understanding of lung tumorigenesis, and hence provide potential diagnostic biomarkers for the malignancy.^20^ For instance, determination of miRNA gene methylation status could be used as a molecular biomarker to detect cancer at an early stage and predict cancer prognosis.^21^ Furthermore, epigenetic alterations could be reversible, making epigenetic regulation attractive in terms of developing new therapeutic approaches.^22^ Since miRNAs can be inactivated by epigenetic mechanisms, drug‐induced reduction of DNA methylation might be a promising therapeutic strategy.^23^ In this study, by analyzing TCGA‐LUAD data, we identified 125 differentially expressed miRNAs and 145 differentially methylated loci in lung tumor tissues versus normal lung tissues. Interestingly, direct interaction between 22 miRNAs and 53 methylation loci were found, suggesting that the miRNAs might be epigenetically regulated by DNA methylation in tumorigenesis of LUAD. Furthermore, hypermethylation and reduced expression of certain miRNAs and increased expression of the target genes were validated in additional surgically‐resected LUAD tissues.

Downregulation of miR‐132‐3p has been observed in several human malignancies, including lung cancer.^24^, ^25^, ^26^, ^27^ For instance, miR‐132‐3p has been suggested to suppress migration and invasion of lung cancer cells by targeting ZEB2.^26^ However, although the studies^24^, ^25^, ^26^ suggested that dysregulation of miR‐132‐3p might have important functions in tumorigenesis, how it contributes to lung cancer remains unknown. Here, we confirmed that miR‐132‐3p was considerably downregulated in lung tumor tissues and ZEB2 could be a direct and functional target of miR‐132‐3p in LUAD. More importantly, our data suggested that elevated expression of miR‐132‐3p, reduced expression of ZEB2, and increased cell proliferation were observed in lung cancer cells treated with DNA methyltransferase inhibitor. Furthermore, forced expressed miR‐132‐3p in LUAD cells reduced their tumorigenicity in vitro and in vivo. Therefore, altered methylation status of miRNAs could regulate expression of the miRNAs, which in turn upregulates the corresponding targeted genes and cell proliferation. miR‐132‐3p could act as a tumor suppressor gene whose downregulation contributes to the progression of lung cancer by directly targeting ZEB2. ZEB2 is a transcription factor and plays vital roles in the TGF‐β signaling cascade and is involved in multiple cellular functions.^28^ ZEB2 could act as a transcriptional repressor of E‐cadherin and plays a role in epithelial‐mesenchymal transition, which leads to specific morphological and phenotypic alterations in tumor cells during cancer metastasis.^29^ In addition, our molecular and cellular function analysis showed that the abnormally methylated miRNAs were mainly involved in cell‐to‐cell signaling and interaction in airway cells, and further support that the direct interaction between two epigenetic aberrations could have important functions in LUAD.

We also identified another two relationships between the two molecular events. A total of 98 methylation loci were not associated with altered miRNA expressions, whereas 103 miRNAs had no relation with the differentially methylated loci. Interestingly, molecular and cellular functional and pathway analyses suggested the two additional relationships between the molecular changes might contribute to tumorigenesis of LUAD via different pathways. Therefore, our results might gain a deeper understanding of the role of epigenetics in lung cacner.

Some limitations exist in this study. (i) We only investigated the effect of CpG island methylation on miRNA expression. However, there is a mutual regulation loop between miRNAs and DNA methylation in human malignancies.^20^ Since a single miRNA can regulate multiple target genes, miRNAs could also regulate DNA methylation by modulating methylation‐related critical proteins.^20^ In a different study, we are investigating the mechanism, by which aberrant DNA methylation could be regulated by dysregulation of miRNAs in lung tumorigenesis. (ii) The focus of this study is LUAD. However, 85% of lung cancers are NSCLCs, which consist of three main subtypes: LUAD, SCC, and LCC. Our ongoing study is to investigate the relationship between the two epigenetic changes in tumorigenesis of SCC and LCC.

In conclusion, the interaction between miRNA and DNA methylation could have important functions in lung tumorigenesis. miR‐132‐3p‐3p downregulation via DNA methylation could promote tumorigenicity of lung cancer by directly regulating ZEB2. Nevertheless, further investigation of the interaction of the two important epigenetic events is needed to improve our understanding of lung tumorigenesis, and hence provide potential diagnostic and therapeutic and targets of lung cancer.

## Disclosure

The authors declare no conflict of interest.

## Supporting information


**Table S1.** Differentially expressed miRNAs between lung adenocarcinoma tissues versus normal tissues.
**Table S2.** The differentially methylated loci in lung adenocarcinoma tissues versus normal lung tissues.
**Table S3.** Differentially expressed miRNAs between lung adenocarcinoma tissues and normal tissues.
**Table S4** The differentially methylated loci were not associated with expression of the miRNAs.
