# Supplementary material for: Integrated analysis of miRNAs and DNA methylation identifies miR‐132‐3p as a tumor suppressor in lung adenocarcinoma
Source: Thorac Cancer. 2020 Jun 4;11(8):2112–24. doi: 10.1111/1759-7714.13497 (PMC7396385; doi:10.1111/1759-7714.13497)
Supplement: Supplementary file 1 — Table S1. Differentially expressed miRNAs between lung adenocarcinoma tissues versus normal tissues. Table S2. The differentially methylated loci in lung adenocarcinoma tissues versus normal lung tissues. Table S3. Differentially expressed miRNAs between lung adenocarcinoma tissues and normal tissues. Table S4 The differentially methylated loci were not associated with expression of the miRNAs. [file TCA-11-2112-s001.docx]

| **Supplementary table 1.** Differentially expressed miRNAs between lung adenocarcinoma tissues vs. normal tissues | | | |
| --- | --- | --- | --- |
| Gene name | log FC | P-value | Regulated |
| hsa-let-7a-1 | -1.50768 | 2.41E-34 | Down-Regulated |
| hsa-let-7a-2 | -1.50441 | 3.61E-34 | Down-Regulated |
| hsa-let-7a-3 | -1.50229 | 2.79E-34 | Down-Regulated |
| hsa-let-7c | -1.79988 | 1.77E-28 | Down-Regulated |
| hsa-let-7e | -1.06131 | 1.17E-15 | Down-Regulated |
| hsa-let-7f-1 | -1.24516 | 6.29E-13 | Down-Regulated |
| hsa-let-7f-2 | -1.24199 | 7.37E-13 | Down-Regulated |
| hsa-mir-105-2 | 1.46159 | 2.06E-11 | Up-Regulated |
| hsa-mir-106a | 1.38860 | 2.08E-11 | Up-Regulated |
| hsa-mir-1-1 | -1.59367 | 2.61E-15 | Down-Regulated |
| hsa-mir-1-2 | -1.59057 | 1.04E-15 | Down-Regulated |
| hsa-mir-1236 | -1.34126 | 1.22E-13 | Down-Regulated |
| hsa-mir-1247 | -2.12685 | 1.88E-17 | Down-Regulated |
| hsa-mir-1269a | 2.81305 | 3.20E-08 | Up-Regulated |
| hsa-mir-1276 | -1.68679 | 2.46E-17 | Down-Regulated |
| hsa-mir-1287 | 1.05310 | 1.63E-13 | Up-Regulated |
| hsa-mir-1301 | 1.30484 | 1.96E-17 | Up-Regulated |
| hsa-mir-1307 | 1.58235 | 6.18E-25 | Up-Regulated |
| hsa-mir-130b | 1.82497 | 3.04E-30 | Up-Regulated |
| hsa-mir-132 | -1.56757 | 1.43E-17 | Down-Regulated |
| hsa-mir-133a-2 | -1.61539 | 1.07E-18 | Down-Regulated |
| hsa-mir-133b | -1.42439 | 1.41E-29 | Down-Regulated |
| hsa-mir-135b | 2.93440 | 1.77E-32 | Up-Regulated |
| hsa-mir-136 | 1.16176 | 2.02E-08 | Up-Regulated |
| hsa-mir-139 | -1.89718 | 2.42E-28 | Down-Regulated |
| hsa-mir-140 | -1.04365 | 1.61E-22 | Down-Regulated |
| hsa-mir-141 | 2.39947 | 2.14E-45 | Up-Regulated |
| hsa-mir-142 | 2.74256 | 4.77E-35 | Up-Regulated |
| hsa-mir-143 | -1.99169 | 1.16E-27 | Down-Regulated |
| hsa-mir-144 | -2.49877 | 8.46E-23 | Down-Regulated |
| hsa-mir-147b | 1.43010 | 1.67E-15 | Up-Regulated |
| hsa-mir-148a | 1.86894 | 1.46E-29 | Up-Regulated |
| hsa-mir-153-2 | 2.81112 | 9.02E-31 | Up-Regulated |
| hsa-mir-17 | 1.07979 | 2.63E-18 | Up-Regulated |
| hsa-mir-182-3p | 2.74013 | 7.97E-60 | Up-Regulated |
| hsa-mir-183-3p | 2.55661 | 2.35E-48 | Up-Regulated |
| hsa-mir-184 | -2.91230 | 1.90E-32 | Down-Regulated |
| hsa-mir-187 | 1.54169 | 5.56E-07 | Up-Regulated |
| hsa-mir-191 | 1.03260 | 4.73E-15 | Up-Regulated |
| hsa-mir-192 | 1.72478 | 3.09E-08 | Up-Regulated |
| hsa-mir-193b | 1.74132 | 2.45E-16 | Up-Regulated |
| hsa-mir-194-1 | 1.29797 | 9.78E-06 | Up-Regulated |
| hsa-mir-194-2 | 1.17220 | 5.88E-05 | Up-Regulated |
| hsa-mir-195 | -1.53911 | 2.30E-29 | Down-Regulated |
| hsa-mir-196a-5p | 2.89786 | 4.38E-12 | Up-Regulated |
| hsa-mir-196b | 2.15883 | 1.83E-09 | Up-Regulated |
| hsa-mir-199a-2 | 1.39102 | 6.33E-22 | Up-Regulated |
| hsa-mir-199b | 1.44171 | 6.10E-22 | Up-Regulated |
| hsa-mir-19a | 1.73862 | 2.11E-20 | Up-Regulated |
| hsa-mir-19b-1 | 1.41700 | 2.00E-20 | Up-Regulated |
| hsa-mir-200a | 2.30541 | 2.29E-37 | Up-Regulated |
| hsa-mir-200b | 1.18457 | 5.73E-12 | Up-Regulated |
| hsa-mir-203b | 1.28720 | 1.82E-06 | Up-Regulated |
| hsa-mir-205 | 1.94506 | 1.14E-05 | Up-Regulated |
| hsa-mir-20a | 1.66932 | 5.81E-26 | Up-Regulated |
| hsa-mir-20b | 1.00202 | 9.27E-05 | Up-Regulated |
| hsa-mir-21 | 2.90893 | 4.04E-96 | Up-Regulated |
| hsa-mir-210 | 5.32171 | 1.79E-66 | Up-Regulated |
| hsa-mir-217 | 1.40891 | 1.41E-10 | Up-Regulated |
| hsa-mir-218-1 | -1.21602 | 2.86E-16 | Down-Regulated |
| hsa-mir-219-1 | -1.17633 | 7.44E-58 | Up-Regulated |
| hsa-mir-224 | 1.69841 | 1.78E-11 | Up-Regulated |
| hsa-mir-2355 | 1.59036 | 9.09E-24 | Up-Regulated |
| hsa-mir-29b-2 | 1.61949 | 4.19E-26 | Up-Regulated |
| hsa-mir-301a | 2.43967 | 5.67E-35 | Up-Regulated |
| hsa-mir-30a-3p | -1.74220 | 3.41E-21 | Down-Regulated |
| hsa-mir-31 | 2.29485 | 4.09E-12 | Up-Regulated |
| hsa-mir-323b | 1.19549 | 4.50E-07 | Up-Regulated |
| hsa-mir-324 | 1.08741 | 9.80E-15 | Up-Regulated |
| hsa-mir-331 | 1.29999 | 4.07E-17 | Up-Regulated |
| hsa-mir-339 | -1.37663 | 3.55E-43 | Down-Regulated |
| hsa-mir-33a | 1.62173 | 7.26E-19 | Up-Regulated |
| hsa-mir-33b | 1.53560 | 3.12E-18 | Up-Regulated |
| hsa-mir-345 | 1.91555 | 1.89E-22 | Up-Regulated |
| hsa-mir-34a | 1.25627 | 4.63E-20 | Up-Regulated |
| hsa-mir-3607 | 3.01717 | 3.25E-24 | Up-Regulated |
| hsa-mir-3677 | 1.11745 | 2.47E-12 | Up-Regulated |
| hsa-mir-375 | 1.23880 | 1.00E-05 | Up-Regulated |
| hsa-mir-378a | -1.52829 | 7.66E-25 | Down-Regulated |
| hsa-mir-378c | -1.00584 | 1.16E-15 | Down-Regulated |
| hsa-mir-409 | 1.37248 | 4.74E-10 | Up-Regulated |
| hsa-mir-424 | 1.63608 | 4.99E-18 | Up-Regulated |
| hsa-mir-429 | 1.94244 | 1.78E-23 | Up-Regulated |
| hsa-mir-450a-2 | 1.44925 | 1.09E-19 | Up-Regulated |
| hsa-mir-450b | 1.41154 | 1.71E-14 | Up-Regulated |
| hsa-mir-451a | -2.31030 | 1.86E-19 | Down-Regulated |
| hsa-mir-454 | 1.30275 | 4.67E-27 | Up-Regulated |
| hsa-mir-455 | 1.62495 | 6.76E-18 | Up-Regulated |
| hsa-mir-4668 | 1.21022 | 2.15E-22 | Up-Regulated |
| hsa-mir-4677 | 1.18121 | 1.10E-23 | Up-Regulated |
| hsa-mir-4732 | -1.57832 | 1.74E-41 | Down-Regulated |
| hsa-mir-486-5p | -3.61768 | 1.38E-44 | Down-Regulated |
| hsa-mir-490 | -1.11481 | 1.05E-24 | Down-Regulated |
| hsa-mir-503 | 1.85274 | 9.27E-22 | Up-Regulated |
| hsa-mir-505 | 1.10232 | 4.32E-14 | Up-Regulated |
| hsa-mir-516a-1 | -1.43379 | 5.433E-05 | Down-Regulated |
| hsa-mir-539 | 1.29577 | 6.04E-10 | Up-Regulated |
| hsa-mir-542 | 1.24475 | 2.30E-13 | Up-Regulated |
| hsa-mir-548d-1 | 2.64239 | 3.80E-32 | Up-Regulated |
| hsa-mir-548v | 1.05797 | 3.13E-13 | Up-Regulated |
| hsa-mir-577 | 2.04145 | 6.72E-16 | Up-Regulated |
| hsa-mir-584 | -1.33697 | 5.15E-12 | Down-Regulated |
| hsa-mir-590 | 1.73750 | 8.86E-32 | Up-Regulated |
| hsa-mir-592 | 1.05400 | 4.34E-13 | Up-Regulated |
| hsa-mir-616 | 1.37450 | 7.73E-17 | Up-Regulated |
| hsa-mir-625 | 1.23614 | 1.89E-17 | Up-Regulated |
| hsa-mir-628 | 1.40720 | 5.74E-14 | Up-Regulated |
| hsa-mir-629 | 1.22467 | 1.32E-21 | Up-Regulated |
| hsa-mir-653 | 1.31377 | 2.00E-08 | Up-Regulated |
| hsa-mir-675 | 1.17768 | 0.0004338 | Up-Regulated |
| hsa-mir-6892 | -1.33873 | 1.87E-21 | Down-Regulated |
| hsa-mir-708 | 3.14814 | 7.91E-58 | Up-Regulated |
| hsa-mir-7-1 | 1.86626 | 3.98E-43 | Up-Regulated |
| hsa-mir-744 | -1.27673 | 4.33E-13 | Down-Regulated |
| hsa-mir-767 | 1.31377 | 0.0007344 | Up-Regulated |
| hsa-mir-877 | -1.23457 | 7.72E-17 | Down-Regulated |
| hsa-mir-889 | 1.00649 | 3.96E-06 | Up-Regulated |
| hsa-mir-891a | 1.03823 | 7.47E-05 | Up-Regulated |
| hsa-mir-9-2 | 4.45014 | 3.79E-32 | Up-Regulated |
| hsa-mir-92b | 1.57865 | 5.57E-14 | Up-Regulated |
| hsa-mir-93 | 1.48763 | 1.17E-24 | Up-Regulated |
| hsa-mir-9-3 | 4.32488 | 8.65E-31 | Up-Regulated |
| hsa-mir-937 | 1.14783 | 1.27E-08 | Up-Regulated |
| hsa-mir-940 | -2.75454 | 2.00E-08 | Down-Regulated |
| hsa-mir-96 | 2.81756 | 3.10E-58 | Up-Regulated |

| **Supplementary table 2**. The differentially methylated loci in lung adenocarcinoma tissues vs. normal lung tissues | | |
| --- | --- | --- |
| Probes | Methylation status between tumor vs. normal tissues | Log2 fold change |
| cg22881914 | Hypermethylation | 1.687 |
| cg23207990 | Hypermethylation | 1.749 |
| cg24898753 | Hypomethylation | 1.609 |
| cg06038133 | Hypomethylation | 1.869 |
| cg13323752 | Hypermethylation | 1.721 |
| cg08575537 | Hypermethylation | 1.282 |
| cg09643544 | Hypermethylation | 1.745 |
| cg15107670 | Hypermethylation | 1.589 |
| cg26186727 | Hypermethylation | 2.392 |
| cg06958829 | Hypermethylation | 1.576 |
| cg04907257 | Hypermethylation | 1.598 |
| cg21591742 | Hypermethylation | 1.527 |
| cg03958979 | Hypermethylation | 2.276 |
| cg25902889 | Hypermethylation | 1.862 |
| cg22660578 | Hypermethylation | 1.804 |
| cg22341310 | Hypermethylation | 1.641 |
| cg13462129 | Hypermethylation | 1.698 |
| cg11376198 | Hypermethylation | 1.551 |
| cg26316946 | Hypermethylation | 1.679 |
| cg03874199 | Hypermethylation | 1.521 |
| cg23130254 | Hypermethylation | 1.521 |
| cg18702197 | Hypermethylation | 1.442 |
| cg21296230 | Hypermethylation | 2.347 |
| cg22187630 | Hypermethylation | 1.31 |
| cg11525285 | Hypermethylation | 1.289 |
| cg05345286 | Hypermethylation | 1.98 |
| cg01839464 | Hypermethylation | 2.182 |
| cg15191648 | Hypermethylation | 1.71 |
| cg02332525 | Hypermethylation | 1.388 |
| cg08832227 | Hypermethylation | 1.425 |
| cg07307078 | Hypermethylation | 2.328 |
| cg25993718 | Hypermethylation | 1.451 |
| cg12782180 | Hypermethylation | 2.007 |
| cg20404387 | Hypermethylation | 1.334 |
| cg18722841 | Hypermethylation | 1.448 |
| cg08109815 | Hypermethylation | 1.526 |
| cg10303487 | Hypermethylation | 1.836 |
| cg02774439 | Hypermethylation | 1.384 |
| cg00489401 | Hypermethylation | 2.36 |
| cg19456540 | Hypermethylation | 1.412 |
| cg03544320 | Hypermethylation | 1.63 |
| cg14008883 | Hypermethylation | 1.745 |
| cg10883303 | Hypermethylation | 1.851 |
| cg12457773 | Hypermethylation | 1.541 |
| cg12768605 | Hypermethylation | 1.366 |
| cg25720804 | Hypermethylation | 1.494 |
| cg19064258 | Hypermethylation | 1.285 |
| cg04534765 | Hypermethylation | 1.342 |
| cg25942450 | Hypermethylation | 1.494 |
| cg01683883 | Hypermethylation | 2.356 |
| cg18952647 | Hypermethylation | 1.518 |
| cg06675478 | Hypermethylation | 1.372 |
| cg26721264 | Hypermethylation | 1.342 |
| cg21245652 | Hypermethylation | 2.224 |
| cg00891541 | Hypermethylation | 1.425 |
| cg10486998 | Hypermethylation | 1.342 |
| cg15343119 | Hypermethylation | 1.342 |
| cg18081258 | Hypermethylation | 2.47 |
| cg13929328 | Hypermethylation | 1.801 |
| cg04490714 | Hypermethylation | 1.395 |
| cg20291049 | Hypermethylation | 1.405 |
| cg10556064 | Hypermethylation | 1.425 |
| cg00662556 | Hypermethylation | 1.342 |
| cg20792062 | Hypermethylation | 2.296 |
| cg20959866 | Hypermethylation | 1.963 |
| cg08441806 | Hypermethylation | 1.596 |
| cg21376883 | Hypermethylation | 2.659 |
| cg00548268 | Hypermethylation | 1.863 |
| cg12111714 | Hypermethylation | 2.204 |
| cg23710218 | Hypermethylation | 1.755 |
| cg06092815 | Hypermethylation | 1.997 |
| cg07536847 | Hypermethylation | 1.797 |
| cg25484904 | Hypermethylation | 1.991 |
| cg10141715 | Hypermethylation | 1.849 |
| cg10235817 | Hypermethylation | 2.365 |
| cg20616414 | Hypermethylation | 1.966 |
| cg17525406 | Hypermethylation | 1.963 |
| cg03734874 | Hypermethylation | 1.55 |
| cg19885761 | Hypermethylation | 1.496 |
| cg21546671 | Hypermethylation | 1.346 |
| cg14384532 | Hypermethylation | 2.148 |
| cg21233722 | Hypermethylation | 1.966 |
| cg23290344 | Hypermethylation | 1.356 |
| cg18536148 | Hypermethylation | 2.563 |
| cg07621046 | Hypermethylation | 1.375 |
| cg04048259 | Hypermethylation | 2.435 |
| cg10088985 | Hypermethylation | 1.412 |
| cg14458834 | Hypermethylation | 1.346 |
| cg25875213 | Hypermethylation | 2.299 |
| cg02919422 | Hypermethylation | 2.547 |
| cg12880658 | Hypermethylation | 2.619 |
| cg23432345 | Hypermethylation | 1.668 |
| cg07703401 | Hypermethylation | 1.572 |
| cg07533148 | Hypermethylation | 2.427 |
| cg04317399 | Hypermethylation | 2.466 |
| cg08089301 | Hypermethylation | 1.346 |
| cg18335068 | Hypermethylation | 1.884 |
| cg07693270 | Hypomethylation | 2.316 |
| cg01717376 | Hypomethylation | 1.574 |
| cg02538046 | Hypermethylation | 1.885 |
| cg02892624 | Hypomethylation | 1.796 |
| cg02928928 | Hypomethylation | 1.853 |
| cg03000593 | Hypermethylation | 1.784 |
| cg04091325 | Hypermethylation | 1.482 |
| cg04678230 | Hypermethylation | 1.784 |
| cg07032866 | Hypermethylation | 1.563 |
| cg07213830 | Hypomethylation | 2.343 |
| cg07245868 | Hypermethylation | 1.676 |
| cg07435282 | Hypomethylation | 1.598 |
| cg07718444 | Hypomethylation | 1.557 |
| cg08457620 | Hypermethylation | 2.454 |
| cg08771019 | Hypomethylation | 1.874 |
| cg10134527 | Hypomethylation | 1.774 |
| cg10817223 | Hypomethylation | 1.795 |
| cg11618529 | Hypermethylation | 1.876 |
| cg12195211 | Hypermethylation | 1.774 |
| cg12646585 | Hypomethylation | 1.996 |
| cg13027595 | Hypomethylation | 1.873 |
| cg13210403 | Hypomethylation | 1.785 |
| cg14018100 | Hypermethylation | 1.663 |
| cg14096569 | Hypomethylation | 1.807 |
| cg14258935 | Hypomethylation | 1.574 |
| cg14288281 | Hypomethylation | 2.383 |
| cg14288848 | Hypomethylation | 1.576 |
| cg14836313 | Hypomethylation | 1.598 |
| cg16979445 | Hypermethylation | 1.527 |
| cg17224769 | Hypomethylation | 2.276 |
| cg17421062 | Hypomethylation | 1.862 |
| cg17736336 | Hypermethylation | 1.804 |
| cg18144560 | Hypomethylation | 1.641 |
| cg19273253 | Hypomethylation | 1.687 |
| cg19619721 | Hypomethylation | 1.749 |
| cg19837124 | Hypomethylation | 1.609 |
| cg20185718 | Hypomethylation | 1.582 |
| cg20256117 | Hypomethylation | 1.453 |
| cg21330831 | Hypomethylation | 1.263 |
| cg21754201 | Hypermethylation | 1.663 |
| cg22123387 | Hypomethylation | 1.589 |
| cg23803468 | Hypomethylation | 2.454 |
| cg25237720 | Hypermethylation | 1.576 |
| cg25355006 | Hypermethylation | 1.598 |
| cg25954512 | Hypomethylation | 1.397 |
| cg26646118 | Hypomethylation | 2.807 |
| cg27168291 | Hypomethylation | 1.917 |
| cg27368379 | Hypomethylation | 1.695 |

| **Supplementary table 3**. Differentially expressed miRNAs between lung adenocarcinoma tissues and normal tissues | | | |
| --- | --- | --- | --- |
| Gene name | log FC | P-value | Regulated |
| hsa-let-7a-1 | -1.50768 | 2.41E-34 | Down-Regulated |
| hsa-let-7a-2 | -1.50441 | 3.61E-34 | Down-Regulated |
| hsa-let-7a-3 | -1.50229 | 2.79E-34 | Down-Regulated |
| hsa-let-7c | -1.79988 | 1.77E-28 | Down-Regulated |
| hsa-let-7e | -1.06131 | 1.17E-15 | Down-Regulated |
| hsa-let-7f-1 | -1.24516 | 6.29E-13 | Down-Regulated |
| hsa-let-7f-2 | -1.24199 | 7.37E-13 | Down-Regulated |
| hsa-mir-105-2 | 1.46159 | 2.06E-11 | Up-Regulated |
| hsa-mir-106a | 1.38860 | 2.08E-11 | Up-Regulated |
| hsa-mir-1-1 | -1.59367 | 2.61E-15 | Down-Regulated |
| hsa-mir-1-2 | -1.59057 | 1.04E-15 | Down-Regulated |
| hsa-mir-1247 | -2.12685 | 1.88E-17 | Down-Regulated |
| hsa-mir-1269a | 2.81305 | 3.20E-08 | Up-Regulated |
| hsa-mir-1287 | 1.05310 | 1.63E-13 | Up-Regulated |
| hsa-mir-1301 | 1.30484 | 1.96E-17 | Up-Regulated |
| hsa-mir-1307 | 1.58235 | 6.18E-25 | Up-Regulated |
| hsa-mir-130b | 1.82497 | 3.04E-30 | Up-Regulated |
| hsa-mir-133a-2 | -1.61539 | 1.07E-18 | Down-Regulated |
| hsa-mir-133b | -1.42439 | 1.41E-29 | Down-Regulated |
| hsa-mir-135b | 2.93440 | 1.77E-32 | Up-Regulated |
| hsa-mir-136 | 1.16176 | 2.02E-08 | Up-Regulated |
| hsa-mir-139 | -1.89718 | 2.42E-28 | Down-Regulated |
| hsa-mir-140 | -1.04365 | 1.61E-22 | Down-Regulated |
| hsa-mir-141 | 2.39947 | 2.14E-45 | Up-Regulated |
| hsa-mir-142 | 2.74256 | 4.77E-35 | Up-Regulated |
| hsa-mir-144 | -2.49877 | 8.46E-23 | Down-Regulated |
| hsa-mir-148a | 1.86894 | 1.46E-29 | Up-Regulated |
| hsa-mir-153-2 | 2.81112 | 9.02E-31 | Up-Regulated |
| hsa-mir-17 | 1.07979 | 2.63E-18 | Up-Regulated |
| hsa-mir-182-3p | 2.74013 | 7.97E-60 | Up-Regulated |
| hsa-mir-183-3p | 2.55661 | 2.35E-48 | Up-Regulated |
| hsa-mir-184 | -2.91230 | 1.90E-32 | Down-Regulated |
| hsa-mir-187 | 1.54169 | 5.56E-07 | Up-Regulated |
| hsa-mir-191 | 1.03260 | 4.73E-15 | Up-Regulated |
| hsa-mir-192 | 1.72478 | 3.09E-08 | Up-Regulated |
| hsa-mir-193b | 1.74132 | 2.45E-16 | Up-Regulated |
| hsa-mir-194-1 | 1.29797 | 9.78E-06 | Up-Regulated |
| hsa-mir-194-2 | 1.17220 | 5.88E-05 | Up-Regulated |
| hsa-mir-195 | -1.53911 | 2.30E-29 | Down-Regulated |
| hsa-mir-196a-5p | 2.89786 | 4.38E-12 | Up-Regulated |
| hsa-mir-196b | 2.15883 | 1.83E-09 | Up-Regulated |
| hsa-mir-199a-2 | 1.39102 | 6.33E-22 | Up-Regulated |
| hsa-mir-199b | 1.44171 | 6.10E-22 | Up-Regulated |
| hsa-mir-19a | 1.73862 | 2.11E-20 | Up-Regulated |
| hsa-mir-19b-1 | 1.41700 | 2.00E-20 | Up-Regulated |
| hsa-mir-203b | 1.28720 | 1.82E-06 | Up-Regulated |
| hsa-mir-205 | 1.94506 | 1.14E-05 | Up-Regulated |
| hsa-mir-20a | 1.66932 | 5.81E-26 | Up-Regulated |
| hsa-mir-20b | 1.00202 | 9.27E-05 | Up-Regulated |
| hsa-mir-21 | 2.90893 | 4.04E-96 | Up-Regulated |
| hsa-mir-210 | 5.32171 | 1.79E-66 | Up-Regulated |
| hsa-mir-217 | 1.40891 | 1.41E-10 | Up-Regulated |
| hsa-mir-218-1 | -1.21602 | 2.86E-16 | Down-Regulated |
| hsa-mir-224 | 1.69841 | 1.78E-11 | Up-Regulated |
| hsa-mir-2355 | 1.59036 | 9.09E-24 | Up-Regulated |
| hsa-mir-29b-2 | 1.61949 | 4.19E-26 | Up-Regulated |
| hsa-mir-301a | 2.43967 | 5.67E-35 | Up-Regulated |
| hsa-mir-30a-3p | -1.74220 | 3.41E-21 | Down-Regulated |
| hsa-mir-31 | 2.29485 | 4.09E-12 | Up-Regulated |
| hsa-mir-323b | 1.19549 | 4.50E-07 | Up-Regulated |
| hsa-mir-324 | 1.08741 | 9.80E-15 | Up-Regulated |
| hsa-mir-331 | 1.29999 | 4.07E-17 | Up-Regulated |
| hsa-mir-33a | 1.62173 | 7.26E-19 | Up-Regulated |
| hsa-mir-33b | 1.53560 | 3.12E-18 | Up-Regulated |
| hsa-mir-345 | 1.91555 | 1.89E-22 | Up-Regulated |
| hsa-mir-34a | 1.25627 | 4.63E-20 | Up-Regulated |
| hsa-mir-3607 | 3.01717 | 3.25E-24 | Up-Regulated |
| hsa-mir-3677 | 1.11745 | 2.47E-12 | Up-Regulated |
| hsa-mir-378a | -1.52829 | 7.66E-25 | Down-Regulated |
| hsa-mir-378c | -1.00584 | 1.16E-15 | Down-Regulated |
| hsa-mir-409 | 1.37248 | 4.74E-10 | Up-Regulated |
| hsa-mir-424 | 1.63608 | 4.99E-18 | Up-Regulated |
| hsa-mir-429 | 1.94244 | 1.78E-23 | Up-Regulated |
| hsa-mir-450a-2 | 1.44925 | 1.09E-19 | Up-Regulated |
| hsa-mir-450b | 1.41154 | 1.71E-14 | Up-Regulated |
| hsa-mir-451a | -2.31030 | 1.86E-19 | Down-Regulated |
| hsa-mir-454 | 1.30275 | 4.67E-27 | Up-Regulated |
| hsa-mir-455 | 1.62495 | 6.76E-18 | Up-Regulated |
| hsa-mir-4668 | 1.21022 | 2.15E-22 | Up-Regulated |
| hsa-mir-4677 | 1.18121 | 1.10E-23 | Up-Regulated |
| hsa-mir-4732 | -1.57832 | 1.74E-41 | Down-Regulated |
| hsa-mir-486-5p | -3.61768 | 1.38E-44 | Down-Regulated |
| hsa-mir-490 | -1.11481 | 1.05E-24 | Down-Regulated |
| hsa-mir-503 | 1.85274 | 9.27E-22 | Up-Regulated |
| hsa-mir-505 | 1.10232 | 4.32E-14 | Up-Regulated |
| hsa-mir-539 | 1.29577 | 6.04E-10 | Up-Regulated |
| hsa-mir-542 | 1.24475 | 2.30E-13 | Up-Regulated |
| hsa-mir-577 | 2.04145 | 6.72E-16 | Up-Regulated |
| hsa-mir-584 | -1.33697 | 5.15E-12 | Down-Regulated |
| hsa-mir-592 | 1.05400 | 4.34E-13 | Up-Regulated |
| hsa-mir-616 | 1.37450 | 7.73E-17 | Up-Regulated |
| hsa-mir-628 | 1.40720 | 5.74E-14 | Up-Regulated |
| hsa-mir-629 | 1.22467 | 1.32E-21 | Up-Regulated |
| hsa-mir-653 | 1.31377 | 2.00E-08 | Up-Regulated |
| hsa-mir-6892 | -1.33873 | 1.87E-21 | Down-Regulated |
| hsa-mir-708 | 3.14814 | 7.91E-58 | Up-Regulated |
| hsa-mir-7-1 | 1.86626 | 3.98E-43 | Up-Regulated |
| hsa-mir-767 | 1.31377 | 0.0007344 | Up-Regulated |
| hsa-mir-889 | 1.00649 | 3.96E-06 | Up-Regulated |
| hsa-mir-891a | 1.03823 | 7.47E-05 | Up-Regulated |
| hsa-mir-93 | 1.48763 | 1.17E-24 | Up-Regulated |
| hsa-mir-9-3 | 4.32488 | 8.65E-31 | Up-Regulated |
| hsa-mir-96 | 2.81756 | 3.10E-58 | Up-Regulated |

| **Supplementary table 4.** The differentially methylated loci were not associated with expression of the miRNAs | |
| --- | --- |
| Probes | Methylation status between tumor and normal tissues |
| cg00489401 | Hypermethylation |
| cg00548268 | Hypermethylation |
| cg00662556 | Hypermethylation |
| cg00891541 | Hypermethylation |
| cg01683883 | Hypermethylation |
| cg01839464 | Hypermethylation |
| cg02332525 | Hypermethylation |
| cg02774439 | Hypermethylation |
| cg02919422 | Hypermethylation |
| cg03544320 | Hypermethylation |
| cg03734874 | Hypermethylation |
| cg03874199 | Hypermethylation |
| cg03958979 | Hypermethylation |
| cg04048259 | Hypermethylation |
| cg04317399 | Hypermethylation |
| cg04490714 | Hypermethylation |
| cg04534765 | Hypermethylation |
| cg04907257 | Hypermethylation |
| cg05345286 | Hypermethylation |
| cg06038133 | Hypomethylation |
| cg06092815 | Hypermethylation |
| cg06675478 | Hypermethylation |
| cg06958829 | Hypermethylation |
| cg07307078 | Hypermethylation |
| cg07533148 | Hypermethylation |
| cg07536847 | Hypermethylation |
| cg07621046 | Hypermethylation |
| cg07693270 | Hypomethylation |
| cg07703401 | Hypermethylation |
| cg08089301 | Hypermethylation |
| cg08109815 | Hypermethylation |
| cg08441806 | Hypermethylation |
| cg08575537 | Hypermethylation |
| cg08832227 | Hypermethylation |
| cg09643544 | Hypermethylation |
| cg10088985 | Hypermethylation |
| cg10141715 | Hypermethylation |
| cg10235817 | Hypermethylation |
| cg10303487 | Hypermethylation |
| cg10486998 | Hypermethylation |
| cg10556064 | Hypermethylation |
| cg10883303 | Hypermethylation |
| cg11376198 | Hypermethylation |
| cg11525285 | Hypermethylation |
| cg12111714 | Hypermethylation |
| cg12457773 | Hypermethylation |
| cg12768605 | Hypermethylation |
| cg12782180 | Hypermethylation |
| cg12880658 | Hypermethylation |
| cg13323752 | Hypermethylation |
| cg13462129 | Hypermethylation |
| cg13929328 | Hypermethylation |
| cg14008883 | Hypermethylation |
| cg14384532 | Hypermethylation |
| cg14458834 | Hypermethylation |
| cg15107670 | Hypermethylation |
| cg15191648 | Hypermethylation |
| cg15343119 | Hypermethylation |
| cg17525406 | Hypermethylation |
| cg18081258 | Hypermethylation |
| cg18335068 | Hypermethylation |
| cg18536148 | Hypermethylation |
| cg18702197 | Hypermethylation |
| cg18722841 | Hypermethylation |
| cg18952647 | Hypermethylation |
| cg19064258 | Hypermethylation |
| cg19456540 | Hypermethylation |
| cg19885761 | Hypermethylation |
| cg20291049 | Hypermethylation |
| cg20404387 | Hypermethylation |
| cg20616414 | Hypermethylation |
| cg20792062 | Hypermethylation |
| cg20959866 | Hypermethylation |
| cg21233722 | Hypermethylation |
| cg21245652 | Hypermethylation |
| cg21296230 | Hypermethylation |
| cg21376883 | Hypermethylation |
| cg21546671 | Hypermethylation |
| cg21591742 | Hypermethylation |
| cg22187630 | Hypermethylation |
| cg22341310 | Hypermethylation |
| cg22660578 | Hypermethylation |
| cg22881914 | Hypermethylation |
| cg23130254 | Hypermethylation |
| cg23207990 | Hypermethylation |
| cg23290344 | Hypermethylation |
| cg23432345 | Hypermethylation |
| cg23710218 | Hypermethylation |
| cg24898753 | Hypomethylation |
| cg25484904 | Hypermethylation |
| cg25720804 | Hypermethylation |
| cg25875213 | Hypermethylation |
| cg25902889 | Hypermethylation |
| cg25942450 | Hypermethylation |
| cg25993718 | Hypermethylation |
| cg26186727 | Hypermethylation |
| cg26316946 | Hypermethylation |
| cg26721264 | Hypermethylation |
